# The association between eating disorders and mental health: an umbrella review

**DOI:** 10.1186/s40337-022-00725-4

**Published:** 2023-03-27

**Authors:** Eng Joo Tan, Tejeesha Raut, Long Khanh-Dao Le, Phillipa Hay, Jaithri Ananthapavan, Yong Yi Lee, Cathrine Mihalopoulos

**Affiliations:** 1 School of Public Health and Preventive Medicine, Monash University Health Economics Group (MUHEG), Monash University, Melbourne, VIC 3004 Australia; 2 Deakin Health Economics, Institute for Health Transformation, School of Health and Social Development, Deakin University, Burwood, VIC 3125 Australia; 3 Translational Health Research Institute (THRI), School of Medicine, Western Sydney University, Locked Bag 1797, Penrith, NSW 2751 Australia; 4 Camden and Campbelltown Hospital, SWSLHD, Campbelltown, NSW 2560 Australia; 5 Global Obesity Centre, Institute for Health Transformation, School of Health and Social Development, Deakin University, Burwood, VIC 3125 Australia; 6 School of Public Health, The University of Queensland, QLD 4006 Herston, Australia; 7 Policy and Epidemiology Group, Queensland Centre for Mental Health Research, QLD 4076 Wacol, Australia

**Keywords:** Eating disorders, Mental health, Depression, Anxiety, Suicide, Risk factors, Systematic review, Umbrella review

## Abstract

**Objective:**

There have been an increasing number of systematic reviews indicating the association between eating disorders (ED), including its risk factors, with mental health problems such as depression, suicide and anxiety. The objective of this study was to conduct an umbrella review of these reviews and provide a top-level synthesis of the current evidence in this area.

**Method:**

A systematic search was performed using four databases (MEDLINE Complete, APA PyscInfo, CINAHL Complete and EMBASE). The inclusion criteria were systematic reviews (with or without meta-analysis), published in the English language between January 2015 and November 2022. The quality of the studies was assessed using the Joanna Briggs Institute Critical Appraisal tools for use of JBI Systematic reviews.

**Results:**

A total of 6,537 reviews were identified, of which 18 reviews met the inclusion criteria, including 10 reviews with meta-analysis. The average quality assessment score for the included reviews was moderate. Six reviews investigated the association between ED and three specific mental health problems: (a) depression and anxiety, (b) obsessive-compulsive symptoms and (c) social anxiety. A further 3 reviews focused on the relationship between ED and attention deficit hyperactivity disorder (ADHD) while 2 reviews focused on ED and suicidal-related outcomes. The remaining 7 reviews explored the association between ED and bipolar disorders, personality disorders, and non-suicidal self-injury. Depression, social anxiety and ADHD are likely to have a stronger strength of association with ED relative to other mental health problems.

**Discussion:**

Mental health problems such as depression, social anxiety and ADHD were found to be more prevalent among people suffering from eating disorders. Further research is necessary to understand the mechanism and health impacts of potential comorbidities of ED.

**Supplementary Information:**

The online version contains supplementary material available at 10.1186/s40337-022-00725-4.

## Abstract (summary)

This review aimed to investigate the association between eating disorders (ED) and mental health problems. A review of existing systematic reviews was conducted to provide a top-level synthesis of the current evidence in this area. Our review found a total of 18 systematic reviews, which investigated the association between ED and a wide range of mental health problems. These conditions include depression and anxiety, obsessive-compulsive symptoms, attention deficit hyperactivity disorder (ADHD), social anxiety, personality disorders, suicidal-related outcomes, bipolar disorders and non-suicidal self-injury. Depression, social anxiety and ADHD are likely to have a stronger strength of association with ED relative to other mental health problems.

**Supplementary Information:**

The online version contains supplementary material available at 10.1186/s40337-022-00725-4.

## Introduction

Eating disorders (ED) such as anorexia nervosa, bulimia nervosa and binge eating disorders lead to higher physical and psychological morbidity, disabilities, and mortality rates [1]. The prevalence of eating disorder is increasing, with the lifetime prevalence between 3.3 and 18.6% among women and between 0.8 and 6.5% among men [2]. Risk factors such as dieting and body dissatisfaction have been considered predictors of ED onset for many years [3]. Other predisposing factors of ED also include family history of EDs, having close relatives with a mental health problem, personal history of anxiety disorder, and behavioural inflexibility and sociocultural issues such as weight stigma, bullying or teasing and limited social networks [4].

Many studies have linked EDs to various mental health problems. For example, personality disorders can be found in a portion of patients with anorexia nervosa (AN) and bulimia nervosa (BN), and were encountered in the treatment of EDs [5]. Binge eating disorder (BED) has been found to impact mental health problems such as anxiety and depression which worsens health-related quality of life (HRQL) of an individual [6]. In a study of a nationally representative sample of 36,309 adults, all three EDs were associated with more than one comorbid somatic condition, which can range from lifetime mood disorders, anxiety disorders, major depressive disorder and alcohol and drug use disorders [7]. It has been widely recognized that individuals with EDs show higher rates of suicidality, which includes complete suicide, suicidal attempt, and suicidal ideation [8]. The negative perception of body image, a risk factor for ED, has also been linked to depression and obesity [9]. Individuals suffering from anorexia nervosa or bulimia nervosa also exhibit social anxiety disorders, have low self-esteem and more likely to feel nervous about their appearances in public places [10–12].

The significant burden of mental health problems necessitates a more comprehensive understanding of the relationship between mental health and ED. Recent evidence suggested that the burden of mental health problems has increased, with suicide as the second leading cause of death among 15–29 years and the annual global cost of depression and anxiety was estimated to be USD 1 trillion [13]. While previous studies and reviews have investigated the association between EDs and specific mental health problems such as anxiety, depression and substance use disorder, there is no existing review that provides a top-level summary of these associations by using a broader definition of mental health. Consequently, there is a lack of comparative analyses of the various mental health problems and their associations with ED. Addressing this gap in current research can assist researchers and clinicians to develop a suite of interventions that has the most impact on reducing the ED-mental health co-morbidity. In this context, an umbrella review is useful because it allows the findings of existing reviews to be compared and contrasted. Therefore, this umbrella review aims to synthesize contemporary evidence in order to better understand the relationship between eating disorders and various mental health problems across demographic and clinical factors.

## Methods

This review adhered to the Joanna Brigg Institute (JBI) guidelines for umbrella reviews [14] and the PRISMA (Preferred Reporting Items for Systematic Reviews and Meta-Analyses) standards [15]. An ethics exemption for this research was approved by the Deakin University Human Research Ethics Committee (DUHREC) (ref. 202–1030). The protocol was registered with PROSPERO: International Prospective Register of Systematic Reviews (ref. CRD42021232372).

### Search strategies and databases

In consultation with an experienced librarian, a literature search to identify potentially eligible publications was performed by the second author (TR) on 16 November 2020. A second literature search was performed by the first author (EJT) on 8 November 2022 to include potential studies published from 16 November 2020 onwards. Both searches were conducted via the EBSCOhost platform on four databases: MEDLINE Complete, APA PyscInfo, CINAHL Complete and EMBASE. The International Classification of Diseases version 10 (ICD-10) was used to define the mental health problems relevant to this review. For the purpose of this review, the disease category of disorders of psychological development, which included disorders related to speech, language, scholastic skills, motor function and autism were not considered. The search terms used in the study were various combinations of eating disorder keywords (e.g., “anorexi*”) and mental health keywords (e.g., “addiction”) using Boolean operators (or/and). Further details of the search terms can be found in Table S1 in the supplementary information file.

### Inclusion and exclusion criteria

The aim of this umbrella review was to identify reviews of studies that investigated the association between eating disorders and mental health problems. Therefore, reviews that reported the association or consequences of EDs or ED risk factors and mental health problems such as depression, anxiety, substance use disorders were included. The inclusion criteria required studies to be systematic reviews with or without meta-analyses while scoping reviews, narrative reviews, or literatures reviews without quality assessment were excluded. For the purpose of this umbrella review, a study is considered a systematic review if it had a clearly formulated research question, reported systematic and reproducible methods to identify, select and critically appraise relevant research studies. The studies were limited to the general population although there were no age or gender restrictions on the participants. All the articles included in the study were human studies, published in the English language published in peer-reviewed journals within the last seven years i.e. from January 2015 to November 2022. Non-review studies such as cohort, prevalence, case-control or cross-sectional studies were excluded from this review. Reviews with the wrong setting, study design, outcomes or the patient population were excluded. Further details of the inclusion and exclusion criteria can be found in Table S2 in the supplementary information file.

### Identification of relevant studies and data extraction

All studies from the database search results were imported into Endnote and duplicates were removed. The remaining studies were then uploaded to Covidence, an online systematic review management tool, for screening [16]. A two-stage screening process applying the inclusion and exclusion criteria was conducted: (a) title and abstract screening and (b) full-text screening. Both screening processes were done independently by two reviewers (TR, EJT) and any discrepancies were discussed and resolved by the third reviewer (LL). The following data were extracted from reviews that fulfilled the inclusion criteria: year of publication, number of included studies, type of eating disorders or risk factors of eating disorders, mental health problem, presence of meta-analysis component, study design, population description, country and effect size (if available). Data extraction was performed by TR and independently checked by EJT and LL.

### Quality assessment

The bias and quality of the included reviews were assessed using the Joanna Briggs Institute Critical Appraisal tools for systematic reviews (The Joanna Briggs Institute, 2017). The purpose of this appraisal tool is to assess the methodological quality of the included studies and to determine the extent of the possibility of bias in design, conduct and analysis. The tool consists of 11 items (further details are available Table S2 in the supplementary information file) include three choices - “Yes”, “No” and “Unclear”. The total score on the scale is 11.

## Results

**Fig. 1:**
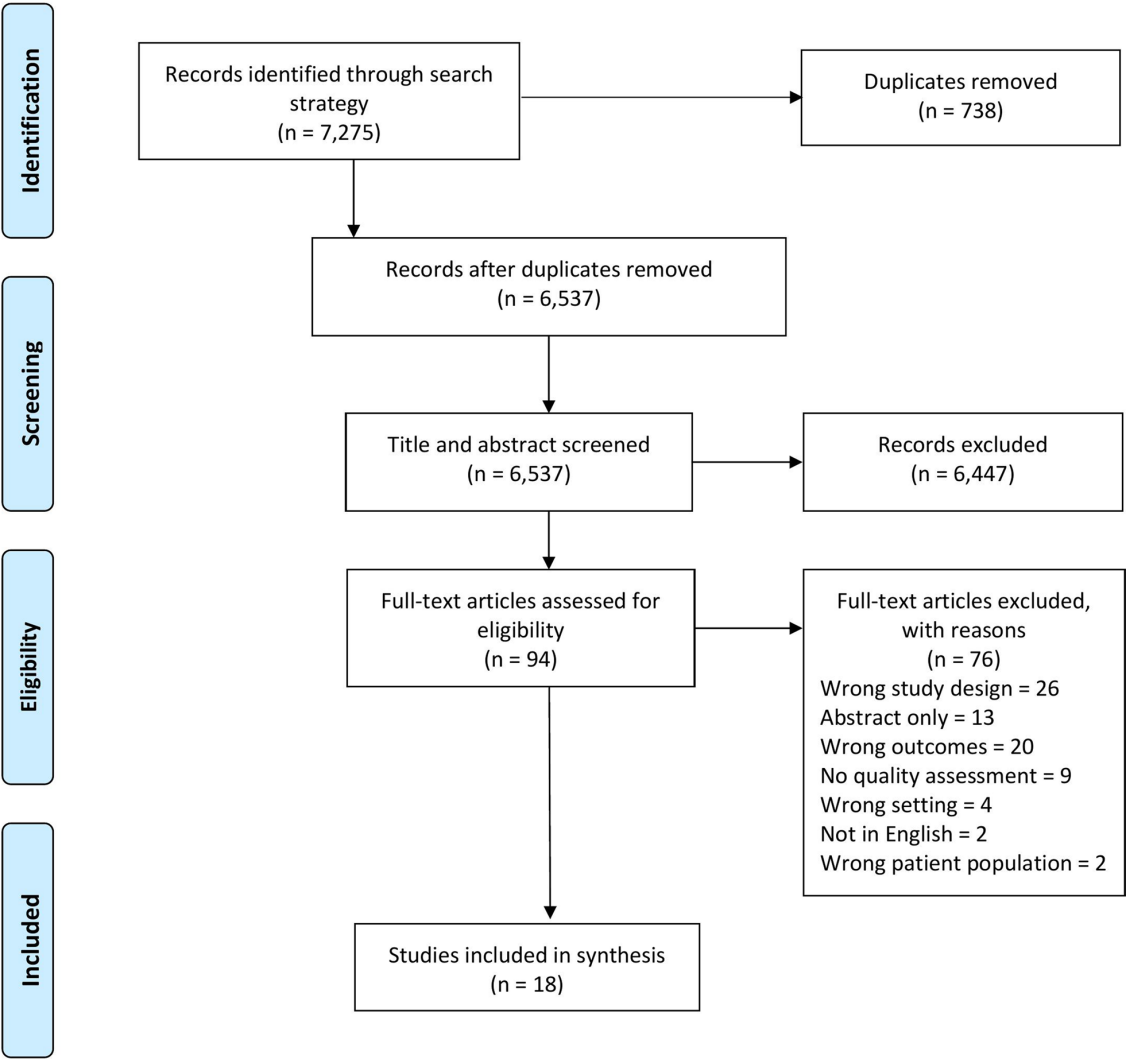
PRISMA flow diagram of included studies

A total of 7,275 potentially relevant studies were identified from the database search. After duplicates were removed, 6,537 studies were available for screening. After title and abstract screening, 94 studies were progressed to full-text screening. Full-text screening resulted in 18 studies meeting the inclusion criteria and being included in the umbrella review. The PRISMA diagram shown in Fig. 1 reports the reason for exclusion for the remaining 76 studies with full-text review.

### Characteristics of included studies

Out of the 18 systematic reviews, ten included a meta-analysis component. There were six reviews investigating the association between ED or ED risk factors (e.g. body dissatisfaction) and three specific mental health problems: (a) depression and anxiety, (b) obsessive-compulsive symptoms and (c) social anxiety. Another three reviews focused on the relationship between ED and attention deficit hyperactivity disorder (ADHD) while two reviews focused on ED and suicidal-related outcomes. The remaining seven reviews explored the association between ED and bipolar disorders, personality disorders, and non-suicidal self-injury. Further details of the included studies are presented in Table 1. The number of individual studies included within the reviews ranged from five to 122 studies with the majority of included studies being conducted using a cross-sectional study design. All but one review investigated the general population, including males and females, and the sample size ranged from 1,792 to 2,321,441 participants.

**Table 1:** Summary of included reviews

Author (year)	Type of eating disorder/ risk factors	Mental health problem	Review type	Number of included studies (and study design if available)	Population description (total sample size, age range and sex)	Effect size of meta-analysis (95% CI)	Overall findings	Quality score*
Álvarez Ruiz et al., [25]	Eating disorder (ED) particularly bulimia nervosa and binge eating disorder	Bipolar disorders (BD)	Systematic review	18 studies on ED in patients with BD, 8 studies on BD in patients with ED	General population (*n* = 7,750, age range = not reported, sex = males and females)	N/A	High comorbidity of bipolar disorder and ED, particularly of bulimia nervosa and binge eating disorder. However, further research needed to determine assessment, treatment and disease etiology.	45%
Baskin & Galligan, [17]	Disordered eating	Depressive and anxiety symptoms, obsessive compulsive symptoms	Systematic review	11 prospective cohort and 14 cross sectional / retrospective studies	Pregnant and post-partum period women (*n* = 318,049, age range = not reported, sex = females)	N/A	Strong evidence for association between disordered eating and depression and anxiety symptoms during pregnancy. Limited evidence for association between disordered eating and obsessive-compulsive symptoms during pregnancy for association between disordered eating and depressive symptoms during the post-partum period.	81%
Conti et al., [8]	Binge eating disorder (BED)	Suicidality (i.e. suicidal ideation or attempted and/or committed suicide)	Systematic review	12 cross-sectional studies 5 longitudinal studies	General population (*n* = 71,610, age range = not reported but generally involved adolescents and adults, sex = males and females)	N/A	BED was significantly associated with higher risk of suicidal behaviors (SB) and suicidal ideation (SI). The correlation between BED and suicide risk is important but there was a lack of studies investigating the size impact of BED on suicide risk.	72%
Cucchi et al., [28]	Eating disorders (ED), anorexia nervosa (AN) and bulimia nervosa (BN)	Non-suicidal self-injury (NSSI)	Systematic review with meta-analysis component	29 studies	General population (*n* = 6,575, age range = 16–30 years old, sex = males and females)	Any ED diagnosis: Prevalence of NSSI = 27.3% (23.8–31.0%) AN diagnosis only: Prevalence of NSSI = 21.8% (18.5–25.6%) BN diagnosis only: Prevalence of NSSI = 32.7% (26.9–39.1%)	Lifetime history of NSSI is highly prevalent among adolescents and young adults with ED, and correlates positively with a history of suicidal attempt.	72%
Drakes et al., [31]	Eating disorders	Obsessive-compulsive disorder	Systematic review with meta-analysis component	59 studies	General population (*n* = unclear, age range = 12–60 years, sex = males and females)	Aggregate lifetime and current prevalence of obsessive-compulsive disorder was 13.9% [95% CI 10.4, 18.1] and 8.7% [95% CI 5.8, 11.8] respectively across EDs	Obsessive-compulsive disorder is prevalent among individuals with a primary diagnosis of eating disorder.	72%
Farstad et al., [29]	Eating disorders, including anorexia nervosa (AN), bulimia nervosa (BN)	Personality disorders (PDs)	Systematic review with meta-analysis component	14 studies	General population (n*n*= 1,884, age range = not reported, sex = males and females)	Pooled prevalence rates ranged from 0% (0–4%) (schizoid) to 30% (0–56%) (obsessive-compulsive) in individuals with ED	Avoidant and obsessive-compulsive PDs were associated with restricting AN and binge-eating disorder while borderline and paranoid PDs were associated with binge-eating/purging AN, BN and other EDs.	63%
Fornaro et al., [26]	Eating disorders, including anorexia nervosa (AN), bulimia nervosa (BN) and binge eating disorder (BED)	Bipolar disorder (BD)	Systematic review with meta-analysis component	47 studies	General population (*n* = 15,146, age range = not reported, sex = males and females)	BED occurred in 12.5% (95%C.I.=9.4–16.6%) of BD cases. BD occurred in 9.1% (95%C.I.=3.3–22.6%) of BED cases. BN occurred in 7.4% (95%C.I.=6–10%) of BD cases. BD occurred in 6.7% (95%C.I.=12-29.2%) of BN cases. AN occurred in 3.8% (95%C.I.=2–6%) of BD cases. BD occurred in 2% (95%C.I.=1–2%) of AN cases.	The comorbidity between ED and BD was present in a considerable number of patients.	72%
Goldstein & Gvion, [27]	Anorexia nervosa (AN) and bulimia nervosa (BN)	Suicidality (i.e. suicidal ideation or attempted and/or death by suicide)	Systematic review	36 cross sectional studies 2 longitudinal studies	General population (n*n*= 2,321,441, age range = not reported, sex = males and females)	N/A	AN and BN were associated with an increased risk of suicidal behaviours and ideation.	63%
Kaisari et al., [22]	Disordered eating behavior	Attention Deficit Hyperactivity Disorder (ADHD)	Systematic review	72 studies including 37 cross sectional studies, 11 case-control studies, 6 cohort studies, 7 longitudinal studies, 2 secondary analysis of the National Longitudinal study of Adolescent Health, 3 experimental studies, 3 retrospective studies, 5 prospective studies and 1 epidemiological study	General population (*n* = 115,418, age range = unclear but includes children, adolescents and adults, sex = males and females)	N/A	Positive association between ADHD and disordered eating. Impulsivity symptoms of ADHD were positively associated with overeating in anorexia nervosa and bulimia nervosa. Further research is needed to determine the direction of relationship and underlying mechanisms.	90%
Kerr-Gaffney et al. [11]	Eating disorders (ED), including anorexia nervosa (AN) and bulimia nervosa (BN)	Social anxiety disorder (SA)	Systematic review with meta-analysis component	38 cross-sectional studies, 12 included in meta-analysis	General population (n = 8,501, age range = 12–45 years old, sex = males and females)	AN diagnosis: *d* = 1.65 (1.03–2.27) BN diagnosis: *d*= 0.71 (0.47–0.95)	Significant differences of AN and BN between ED groups and healthy controls. High levels of SA are associated with more severe form of ED.	81%
Levin & Rawana, [12]	Eating disorders (ED), including disordered eating, anorexia nervosa (AN), bulimia nervosa (BN) and binge eating disorder (BED)	Attention-deficit/hyperactivity disorder (ADHD)	Systematic review	37 studies, including 27 cross sectional studies.	General population (*n* = 74,852 participants, age range = 5–49 years old, sex = males and females)	N/A	Childhood ADHD increases the risk of disordered eating or developing ED in later life.	72%
Lloyd et al., [18]	Anorexia Nervosa (AN)	Anxiety	Systematic review	8 studies, including 4 retrospective case control studies and 4 prospective cohort studies	General population (*n* = 1,670,312, age range = unclear, sex = males and females)	N/A	Anxiety disorder diagnosis in general may predict increased anorexia nervosa risk. However, longitudinal associations between specific anxiety disorders and subsequent AN onset unclear.	81%
Mandelli et al., [32]	Eating disorders, including anorexia nervosa (AN), bulimia nervosa (BN) and binge eating disorder (BED)	Obsessive-compulsive disorder	Systematic review with meta-analysis component	32 studies	General population (n = unclear, mean age range = 15–45 years, sex = males and females	Lifetime and current comorbidity rates: 19% and 14% in AN patients; 13% and 9% in BN patients. Higher lifetime estimates based on prospective follow up studies: 44% in AN patients; 19% in BN patients.	OCD comorbidity in EDs is a significant phenomenon, affecting almost one fifth of the patients in cross-sectional observations and up to nearly 40% in prospective follow-up studies.	81%
Miller et al., [30]	Eating disorders, including anorexia nervosa (AN), bulimia nervosa (BN) and binge eating disorder (BED)	Border personality disorder (BPD) symptoms	Systematic review with meta-analysis component	122 studies	General population (*n* = unclear, age range = 12 years and above, sex = males and females	Affective instability was the BPD symptom most elevated, while anger was the BPD symptom least elevated, in patients with EDs compared to controls.	Nine symptoms of borderline personality disorder were significantly elevated in patients with EDs compared to controls. Certain symptoms of BPD play a more prominent role in the comorbidity between BPD and EDs than others.	
Nazar et al., [24]	Eating disorders (ED), including anorexia nervosa (AN), bulimia nervosa (BN) and binge eating disorder (BED)	Attention-Deficit/Hyperactivity disorder (ADHD)	Systematic review with meta-analysis component	17 studies	General population (*n* = 38,421, age range = 9–44 years old, sex = males and females)	Pooled effect ED diagnosis in ADHD: OR = 3.82 (2.34–6.24) AN diagnosis in ADHD: OR = 4.28 (2.24–8.16) BN diagnosis in ADHD: OR = 5.71 (3.56–9.16) BED diagnosis in ADHD: OR = 4.13 (3-5.67) ADHD diagnosis in ED: OR = 2.57 (1.30–5.11)	The risk having an ED for individuals with ADHD is increased three-fold and the risk of having ADHD for individuals with ED is increased by two-fold.	90%
Nicholls et al., [19]	Binge eating disorder (BED)	Emotions and eating behavior	Systematic review	15 studies, with 13 studies reported on adults and 2 studies reported on children	General population (*n* = 2,858, age range = 10–47 years old, sex = males and females)	N/A	Depression was consistently associated with binge eating. Negative mood was found to be an antecedents of binge eating within an adult BED-obese sample. However, findings were mixed regarding the role of stress, anger, and positive emotions.	72%
Puccio et al., [20]	Eating pathology	Depression	Systematic review with meta-analysis component	42 studies assessing longitudinal relationship between eating pathology and depression	General population (*n* = 73,115, age range = 6–50 years old, sex = males and females)	Correlation value for eating pathology on depression = 0.13 (0.09–0.17) with p < 0.001 Correlation value for depression predicting eating pathology = 0.16 (0.10–0.22), p < 0.001.	Eating pathology is one of the risk factors for depression and vice-versa.	63%
Silva et al., [9]	Body image	Depression	Systematic review	5 cross-sectional studies	General population (*n* = 35,518, age range = 18 years and older, sex = males and females)	N/A	Depression or depressive symptoms were associated with body image for both men and women.	63%

SMD = standardized mean difference; OR = odds ratio

*The quality score was calculated from the total score out of 11 based on the Joanna Briggs Institute (JBI) Critical Appraisal Checklist for Systematic Reviews questionnaire

### ED, depression and anxiety, obsessive compulsive symptoms and social anxiety

The evidence from two reviews [17–19] suggest that individuals afflicted with BED or disordered eating have a higher risk of experiencing negative mood, tension, sadness and emotional instability [19], which can further develop into depressive and anxiety symptoms [17]. However, limited evidence was found to support any link between disordered eating and obsessive-compulsive symptoms [17]. There is evidence to suggest that the relationship between anxiety and AN can be bi-directional. For example, the review by Lloyd et al. [18] demonstrated that the risk of anorexia is predicted to increase in adolescents and young adults diagnosed with an anxiety disorder. Meanwhile, Kerr-Gaffney et al. [11] conducted a systematic review and meta-analysis and found that both BN and AN were associated with social anxiety with a medium effect size of 0.71 [95% CI 0.47, 0.95; *p* < 0.001] and a large effect size of 1.65 [95% CI 1.03, 2.27; *p* < 0.001], respectively as estimated using the Cohen’s d statistic. The authors concluded that individuals with AN or BN have high levels of social anxiety compared to healthy controls.

Several reviews have indicated that certain ED risk factors can potentially contribute to depression. The systematic review and meta-analysis conducted by Puccio et al. [20] suggested that eating pathology is one of the risk factors for depression and vice-versa. The effect of eating pathology on depression among 18,641 females aged 6–50 years was shown to be significant with an effect size of 0.13 (95% CI: 0.09 to 0.17, *p* < 0.001), which was conducted on r values [19]. A systematic review of body image dissatisfaction and depression found that in men the perception of being underweight or dissatisfaction due to low weight was observed by idealizing a larger body, whereas women perceived their body larger than it was by idealizing a lean body [21]. Both of these conditions were associated with the presence of depression or depressive symptoms although the review was unable to conclude whether more severe body image dissatisfaction increased chances of also having depressive symptoms or both conditions co-exist.

### ED and attention deficit hyperactivity disorder

A systematic review conducted by Kaisari et al. [22] on disordered eating behaviour and (ADHD) among 115,418 participants (including both male and female populations) suggested that the impulsivity symptoms of ADHD were positively associated with overeating in AN and BN. Similarly, Levin & Rawana [23] explored the association between AN, BN and BED and ADHD among 74,852 participants and showed that childhood ADHD increases the risk of disordered eating or developing ED in later life. The systematic and meta-analysis of ED on ADHD by Nazar et al. [24] showed that the pooled odds ratio of diagnosing any ED in ADHD populations was 3.82 (95% CI 2.34–6.24). BN has the highest odds ratio (5.71, 95% CI 3.56–9.16) followed by AN (4.28, 95% CI 2.24–8.16) and BED (4.13, 95% CI 3.00–5.67). On the other hand, the pooled odds ratio of diagnosing ADHD in people with eating disorders was 2.57 (95% CI 1.30–5.11) [24].

### ED and bipolar disorder

The systematic review by Álvarez Ruiz & Gutiérrez-Rojas [25] found that the severity of BN and BED in women was higher among patients with bipolar disorder. The evidence from their review suggested that there is a comorbidity between ED and bipolar disorder, with prevalence rate of EDs in bipolar disorder patients ranging from 5.3 to 31%. In addition, a more recent meta-analytic review of 47 studies reported the lifetime prevalence of AN, BN and BED as 3.8% (95% CI 2–6%), 7.4% (95% CI 6–10%) and 12.5% (95% CI 9.40–16.6%) among individuals with bipolar disorder, respectively [26].

### ED and suicidal factors

A systematic review of 12 cross-sectional and 5 longitudinal studies on BED and suicidal factors among adolescents and adults found that BED is associated with a higher risk of suicide, including suicidal behaviours and ideation [8]. Similarly, the systematic review by Goldstein & Gvion [27], which included 36 cross-sectional studies and 2 longitudinal studies, suggested that eating disorders with purging behaviour, impulsivity and specific interpersonal features were associated with greater risk of suicidal behaviours.

### ED and non-suicidal self-injury

A systematic review and meta-analysis by Cucchi et al. [28] reported that, among patients with various EDs, the prevalence of a lifetime history of non-suicidal self-injury (NSSI) was 27.3% (95% CI 23.8–31.0%) for ED, 21.8% (95% CI 18.5–25.6%) for AN, and 32.7% (95% CI 26.9–39.1%) for BN. Based on 29 studies and 6,575 participants, the review concluded that NSSI is a significant correlate of ED and prevalent among adolescents and young adults with ED.

### ED and personality disorders

The systematic review and meta-analysis conducted by Farstad et al. [29] on ED and personality disorders (PD) included 14 studies and showed that pooled prevalence rates of PD ranged from 0% (95% CI: 0–4%) (for schizoid) to 30% (95% CI 0–56%) (for obsessive-compulsive) in individuals with ED. The authors concluded that increases in perfectionism, neuroticism, low extraversion, sensitivity to social rewards, avoidance motivation, negative urgency and high-self-directedness was found in the people presenting with EDs. This finding is consistent with another review that investigated the association between EDs and symptoms of borderline personality disorder [30]. The authors found that nine symptoms of borderline personality disorder were significantly elevated in patients with EDs compared to controls.

In a meta-analytic review of 59 studies, the lifetime and current prevalence of obsessive-compulsive disorder was reported to be 13.9% [95% CI 10.4–18.1%] and 8.7% [95% CI 5.8–11.8%] respectively across EDs, which included all ED subtypes [31]. Another meta-analysis review reported lifetime comorbidity rates for obsessive-compulsive disorder of 19% in AN patients and 14% in BN patients based on cross-sectional studies [32]. These rates increased to 44% in AN patients and 18.5% in BN patients when longitudinal studies were considered.

### Quality of included systematic reviews

The scores achieved by the included reviews ranged from 45% (i.e. 5 out of 11 questions) to 100% (i.e. 11 out of 11 questions). On average, the reviews met 72% of the JBI criteria. The details of the score are presented in Table S3 in the supplementary information file. Overall quality was acceptable and most reviews performed well in the design of review question, inclusion criteria, search strategy and criteria used for study appraisal. The main loss of scores were from the criteria of methods to minimize errors in data extraction and assessment of publication bias.

## Discussion

To the best of our knowledge, this is the first umbrella review to examine the overall evidence of the association between eating disorders and mental health across the age spectrum. While previous reviews were focused on investigating the relationship between eating disorders and specific mental health problems, our review captured all relevant mental health problems, including mental disorders, personality disorders and suicide-related outcomes. The findings of this review were synthesized from contemporaneous systematic reviews (i.e. in the last 7 years) and highlighted the growing body of evidence in this area, particularly the frequency of comorbidity of ED and mental health problems. In addition, our review provides a top-level summary of the strength of the association between the various mental health problems and eating disorders, and the direction of effect where possible.

A total of 643 individual studies were reviewed by the 18 systematic reviews included in this umbrella review. The synthesis of evidence revealed that there is a significant association between ED and mental health problems in general. However, among the various mental health problems investigated, only reviews focusing on depression, social anxiety and ADHD reported an effect size or odds ratio from their respective meta-analysis. Therefore, based on quantitative evidence, the association between these three mental health problems and ED is more prominent compared to other mental health problems. There is also evidence to suggest that depression and anxiety are significantly associated with different types of EDs and their risk factors. For example, symptoms of depression and anxiety were often observed in individuals suffering from AN, BN and BED or those with ED risk factors such as body dissatisfaction [16, 21]. Interestingly, existing research shows that childhood ADHD increased the risk of disordered eating or developing ED in later life and vice versa while the risk of ADHD in individuals with ED is increased three-fold, compared to control groups [24]. This phenomenon is particularly relevant for prevention efforts given that diagnosis of ADHD in young girls or women can be delayed or missed [33]. As such, there are potential shared benefits to be gained when addressing both conditions. Further research is required to explore the underlying mechanisms and comorbidity between EDs and mental disorders. The prevention or treatment of this comorbidity also needs to be addressed by future intervention studies.

While females continue to be disproportionately affected by ED, including through its association with other mental health problems, there is also growing evidence to indicate the adverse impacts of the ED-mental disorder comorbidity on the male population. For example, the correlation between the risk of developing eating pathology due to childhood ADHD was observed to be stronger in males compared to females [23]. Furthermore, restrictive eating behaviour has been linked to ADHD-related hyperactivity symptoms in boys although the causal pathway is still not fully understood [34, 35] As the population group investigated by the reviews included in this study was predominantly females, the association between ED and mental health may be underestimated in males. A balanced representation of the two sexes should be considered in future studies and will lead to an improved understanding of the function of gender in this emerging comorbidity.

Our umbrella review also reported that most of the research were undertaken in high-income countries, whereas limited studies have been conducted in low- and middle-income countries. This is not surprising given that previous evidence have indicated a severe scarcity of mental health research resources in low- and middle-income countries, especially in Asian and African countries [36]. Furthermore, ED-related epidemiology research in low- and middle-income countries often focused on prevalence studies and less on comorbidity between ED and mental health problems [37]. Therefore, there is a need to address this gap in the literature and investigate the generalizability of present evidence across different regions.

One of the limitations of our umbrella review is that it did not include reviews published in languages other than English. In addition, our literature search was limited to the last 7 years, therefore, reviews published before 2015 were not considered. However, it is likely that the more recent reviews in our study have included previous evidence. Another limitation is that no recent individual studies were included. Although this omission may have an impact on the findings of our study, it is unlikely to change the overall conclusion.

Overall, there may be several clinical implications from our findings. First, there is a need to increase awareness and screening for ED in general mental health settings and broader demographics. Compared to general mental health, ED is often underdiagnosed in primary care and therefore the health burden of ED is largely hidden even though it is substantial [38, 39]. Second, it is necessary to address the unmet need for treatment of ED. Evidence has shown that although a majority of community cases with a diagnosable ED who seek treatment received treatment for weight loss, only a small proportion received appropriate mental health care [40]. There is a need to promote supported integrated treatments such as the introduction of mood intolerance module in temperament based therapy with supports [41].

## Conclusion

The outcome of the umbrella review suggests that eating disorders and mental health problems are significantly associated with each other. Mental health problems such as depression, anxiety, suicidal attempts are found to be more prevalent among people suffering from eating disorders. EDs also arise from impulsive behaviours, poor emotion regulation, history of childhood physical and emotional abuse, pain tolerance and interpersonal fears such as perceived burdensomeness [16, 27]. Our findings suggest that there is a need for further research to understand the health impacts of eating disorder and mental disorder comorbidities. For instance, there is a limited assessment of risk factors of suicide in people with ED and, therefore, historical and contemporary data need to be collected in order to better understand the risk of suicide in ED. Further efforts should also be made to identify effective and cost-effective interventions for the prevention or treatment of ED and its comorbidities.

## Supplementary Information

**Additional file 1: Table S1: **Search terms. **Table S2:** Inclusion & exclusion criteria. **Table S3:** The Joanna Briggs Institute Critical Appraisal tools for use of JBI Systematic Reviews-Questionnaires

## Data Availability

All relevant data are within the manuscript and supplementary materials.

## References

[1] Treasure J, Duarte TA, Schmidt U (2020). Eating disorders. The Lancet.

[2] Galmiche M, Déchelotte P, Lambert G, Tavolacci MP (2019). Prevalence of eating disorders over the 2000–2018 period: a systematic literature review. Am J Clin Nutr.

[3] Le LK-D, Barendregt JJ, Hay P, Mihalopoulos C (2017). Prevention of eating disorders: a systematic review and meta-analysis. Clin Psychol Rev.

[4] National eating disorders association. 2020, ‘Risk factors of eating disorders’, NEDA feeding hope, retrieved on 2 February 2021; https://www.nationaleatingdisorders.org/risk-factors.

[5] Martinussen M, Friborg O, Schmierer P, Kaiser S, Øvergård KT, Neunhoeffer A-L (2017). The comorbidity of personality disorders in eating disorders: a meta-analysis. Eating and weight disorders - studies on Anorexia. Bulimia  Obesity.

[6] Singleton C, Kenny TE, Hallett D, Carter JC. Depression partially mediates the Association between binge eating disorder and health-related quality of life. Front in Psychol. 2019;10(209). 10.3389/fpsyg.2019.00209 PMC6399201 30863331

[7] Udo T, Grilo CM (2019). Psychiatric and medical correlates of DSM-5 eating disorders in a nationally representative sample of adults in the United States. Int J Eat Disord.

[8] Conti C, Lanzara R, Scipioni M, Iasenza M, Guagnano MT, Fulcheri M. The relationship between binge eating disorder and suicidality: a systematic review. Front Psychol. 2017;8(2125). 10.3389/fpsyg.2017.02125 PMC5723427 29259574

[9] Silva D, Ferriani L, Viana MC (1992). Depression, anthropometric parameters, and body image in adults: a systematic review. Rev Assoc Med Bras.

[10] Ciarma JL, Mathew JM (2017). Social anxiety and disordered eating: the influence of stress reactivity and self-esteem. Eat Behav.

[11] Kerr-Gaffney J, Harrison A, Tchanturia K (2018). Social anxiety in the eating disorders: a systematic review and meta-analysis. Psychol Med.

[12] Levinson CA, Brosof LC, Vanzhula I, Christian C, Jones P, Rodebaugh TL, Langer JK, White EK, Warren C, Weeks JW, Menatti A (2018). Social anxiety and eating disorder comorbidity and underlying vulnerabilities: using network analysis to conceptualize comorbidity. Int J Eating Disorders.

[13] World Health Organization (WHO). ‘Mental Health’, World Health Organization, retrieved 11 February 2021; https://www.who.int/health-topics/mental-health#tab=tab_2.

[14] The Joanna Briggs Institute. 2017, ‘Checklist for systematic reviews and research syntheses’, The Joanna Briggs Institute, retrieved on 13 Jan 2021; https://joannabriggs.org/sites/default/files/2019-05/JBI_Critical_Appraisal-Checklist_for_Systematic_Reviews2017_0.pdf.

[15] Page MJ, McKenzie JE, Bossuyt PM, Boutron I, Hoffmann TC, Mulrow CD (2021). The PRISMA 2020 statement: an updated guideline for reporting systematic reviews. BMJ.

[16] Veritas Health Innovation (2020). Covidence systematic review software Melbourne.

[17] Baskin R, Galligan R (2019). Disordered eating and the perinatal period: a systematic review and best evidence synthesis of mental health and psychosocial correlates. Eur Eat Disorders Rev.

[18] Lloyd EC, Haase AM, Foster CE, Verplanken B (2019). A systematic review of studies probing longitudinal associations between anxiety and anorexia nervosa. Psychiatry Res.

[19] Nicholls W, Devonport TJ, Blake M (2016). The association between emotions and eating behaviour in an obese population with binge eating disorder. Obes Rev.

[20] Puccio F, Fuller-Tyszkiewicz M, Ong D, Krug I (2016). A systematic review and meta-analysis on the longitudinal relationship between eating pathology and depression. Int J Eat Disord.

[21] Rosenthal R, DiMatteo MR (2001). Meta-analysis: recent developments in quantitative methods for literature reviews. Ann Rev Psychol.

[22] Kaisari P, Dourish CT, Higgs S (2017). Attention deficit hyperactivity disorder (ADHD) and disordered eating behaviour: a systematic review and a framework for future research. Clin Psychol Rev.

[23] Levin RL, Rawana JS (2016). Attention-deficit/hyperactivity disorder and eating disorders across the lifespan: a systematic review of the literature. Clin Psychol Rev.

[24] Nazar BP, Bernardes C, Peachey G, Sergeant J, Mattos P, Treasure J (2016). The risk of eating disorders comorbid with attention-deficit/hyperactivity disorder: a systematic review and meta-analysis. Int J Eat Disord.

[25] Álvarez Ruiz EM, Gutiérrez-Rojas L (2015). Comorbidity of bipolar disorder and eating disorders. Revista de Psiquiatría y Salud Mental (English Edition).

[26] Fornaro M, Daray FM, Hunter F, Anastasia A, Stubbs B, De Berardis D (2021). The prevalence, odds and predictors of lifespan comorbid eating disorder among people with a primary diagnosis of bipolar disorders, and vice-versa: systematic review and meta-analysis. J Affect Disorders.

[27] Goldstein A, Gvion Y (2019). Socio-demographic and psychological risk factors for suicidal behavior among individuals with anorexia and bulimia nervosa: a systematic review. J Affect Disord.

[28] Cucchi A, Ryan D, Konstantakopoulos G, Stroumpa S, Kaçar A, Renshaw S (2016). Lifetime prevalence of non-suicidal self-injury in patients with eating disorders: a systematic review and meta-analysis. Psychol Med.

[29] Farstad SM, McGeown LM, von Ranson KM (2016). Eating disorders and personality, 2004–2016: a systematic review and meta-analysis. Clin Psychol Rev.

[30] Miller AE, Trolio V, Halicki-Asakawa A, Racine SE (2022). Eating disorders and the nine symptoms of borderline personality disorder: a systematic review and series of meta-analyses. Int J Eat Disord.

[31] Drakes DH, Fawcett EJ, Rose JP, Carter-Major JC, Fawcett JM (2021). Comorbid obsessive-compulsive disorder in individuals with eating disorders: an epidemiological meta-analysis. J Psychiatr Res.

[32] Mandelli L, Draghetti S, Albert U, De Ronchi D, Atti A-R (2020). Rates of comorbid obsessive-compulsive disorder in eating disorders: a meta-analysis of the literature. J Affect Disord.

[33] Hinshaw SP, Nguyen PT, O’Grady SM, Rosenthal EA (2022). Annual research review: attention-deficit/hyperactivity disorder in girls and women: underrepresentation, longitudinal processes, and key directions. J Child Psychol Psychiatry.

[34] Gaub M, Carlson CL (1997). Gender differences in ADHD: a meta-analysis and critical review. J Am Acad Child Adolesc Psychiatry.

[35] Williamson D, Johnston C (2015). Gender differences in adults with attention-deficit/hyperactivity disorder: a narrative review. Clin Psychol Rev.

[36] Razzouk D, Sharan P, Gallo C, Gureje O, Lamberte EE, de Jesus Mari J (2010). Scarcity and inequity of mental health research resources in low-and-middle income countries: a global survey. Health Policy.

[37] Hoek HW (2016). Review of the worldwide epidemiology of eating disorders. Curr Opin Psychiatry.

[38] Mond JM, Myers TC, Crosby RD, Hay PJ, Mitchell JE (2010). Bulimic eating disorders in primary care: hidden morbidity still?. J Clin Psychol Med Settings.

[39] Fursland A, Watson HJ (2014). Eating disorders: a hidden phenomenon in outpatient mental health?. Int J Eat Disord.

[40] Hart LM, Granillo MT, Jorm AF, Paxton SJ (2011). Unmet need for treatment in the eating disorders: a systematic review of eating disorder specific treatment seeking among community cases. Clin Psychol Rev.

[41] Fairburn CG. Cognitive behavior therapy and eating disorders. US: Guilford Press; 2008. xii, 324-xii, p.

