# Supplementary material for: The association between eating disorders and mental health: an umbrella review
Source: J Eat Disord. 2023 Mar 27;11:51. doi: 10.1186/s40337-022-00725-4 (PMC10044389; doi:10.1186/s40337-022-00725-4)
Supplement: Supplementary file 1 — Additional file 1: Table S1: Search terms. Table S2: Inclusion & exclusion criteria. Table S3: The Joanna Briggs Institute Critical Appraisal tools for use of JBI Systematic Reviews-Questionnaires [file 40337_2022_725_MOESM1_ESM.docx]

**Supplementary Information**

**The association between eating disorders and mental health:**

**An umbrella review**

Short running title: A review of eating disorders and mental health

Eng Joo Tan^1^, Tejeesha Raut^2^, Long Khanh-Dao Le^1^, Phillipa Hay ^3,4^, Jaithri Ananthapavan^2,5^, Yong Yi Lee^1,6,7^, Cathrine Mihalopoulos^1^

^1^ Monash University Health Economics Group (MUHEG), School of Public Health and Preventive Medicine, Monash University, Melbourne, VIC 3004, Australia (EJ Tan PhD, L Le PhD, J Ananthapavan PhD, YY Lee PhD, Prof C Mihalopoulos PhD)

^2^ Deakin Health Economics, Institute for Health Transformation, School of Health and Social Development, Deakin University, Burwood, Victoria 3125, Australia (T Raut MHE, J Ananthapavan PhD)

^3^ Translational Health Research Institute (THRI), School of Medicine, Western Sydney University, Locked Bag 1797, Penrith, NSW 2751, Australia (Prof P Hay MD, DPhil)

^4^ Camden and Campbelltown Hospital, SWSLHD, Campbelltown, NSW 2560, Australia (Prof P Hay MD, DPhil)

^5^ Global Obesity Centre, Institute for Health Transformation, School of Health and Social Development, Deakin University, Burwood, Victoria 3125, Australia (J Ananthapavan PhD)

^6^ School of Public Health, The University of Queensland, Herston, QLD 4006, Australia (YY Lee PhD)

^7^ Policy and Epidemiology Group, Queensland Centre for Mental Health Research, Wacol, QLD 4076, Australia (YY Lee PhD)

Correspondence to: Dr Eng Joo Tan

Monash University Health Economics Group (MUHG),

School of Public Health and Preventive Medicine, Monash University, Melbourne, VIC 3004, Australia

[andrew.tan1@monash.edu](mailto:andrew.tan1@monash.edu.au)

+61-497524834

**Table S1: Search terms**

| **Title/Abstract (Databases used: MEDLINE Complete, APA PsychInfo, CINAHL Complete & EMBASE)** |
| --- |
| “eating disorder*” |
| “disorder eating” |
| “weight control behavio*” |
| “anorexi*” |
| “bulimi*” |
| ((“shape* or weight or eating) N2 concern*”) |
| (“thin-ideal internali?ation”) |
| (“negative affect*”) |
| (“body dissatisfaction”) |
| “body satisfaction” |
| “body image” |
| “body sha?e” |
| “body sham*” |
| “ideal body” |
| “body type” |
| “body size” |
| body N3 dissatisfaction |
| body N3 satisf* |
| body N3 image |
| body N3 sha?e |
| body N3 sham* |
| ideal N3 body |
| desir* N3 body |
| body N3 type |
| body N3 size |
| purg* |
| “eating pathology*” |
| “pressure to be thin” |
| “negative affect” |
| “thin-ideal” |
| “binge eat*” |
| “mental disorder*” |
| “mental illness*” |
| “mental health” |
| Mental |
| Depression |
| depress* |
| anxi* |
| Bipolar |
| Schizophrenia |
| “Attention Deficit Hyperactivity Disorder*” |
| “Attention Deficit Disorder*” |
| “Oppositional defiant disorder*” |
| Schizoaffective |
| Mania |
| Manic |
| “adjustment disorder*” |
| (somatoform or sleep) |
| (neurotic or neurosis) |
| “gender identit*” |
| hyperkinetic |
| “attachment disorder*” |
| (enuresis OR encopresis) |
| (trichotillomania or Resilience) |
| “social psychology” |
| Positive psychology |
| Stress |
| “coping behavior*” |
| “mental well*” |
| “positive mental health” |
| “coping mechanism” |
| “coping strateg*” |
| “social emotional” |
| loneliness |
| “social isolation” |
| Behavioural disorder |
| Dissociation disorders |
| “Dissociative disorder*” |
| Psychosis |
| “Obsessive Compulsive Disorder” |
| (Delusion* or mood) |
| “Personality Disorder” |
| “Affective Disorder” |
| Phobi* |
| “Borderline Personality Disorder*” |
| “Conduct Disorder” |
| “Emotional abuse” |
| hallucination |
| “Personality disorder*” |
| “Antisocial behavio*” |
| Suicide |
| “Obsessive compulsive disorder*” |
| Panic |
| “Post-traumatic stress disorder*” |
| Psychotic |
| Self-harm |
| “Drug abuse” |
| (suicidal or suicid*) |
| (selfharm* or self-harm*) |
| (self AND harm*) |
| selfinjur* |
| (self-injur* OR (self AND injur*)) |
| (selfpoison* or self-poison*) |
| (self AND poison*) |
| (selfcut* OR self-cut*) |
| (self AND cut*) |
| (parasuicid* OR para-suicid*) |
| (deliberat* OR intent*) |
| overdos* |
| (alcohol or substance*) |
| addiction |
| marijuana |
| dysthymi* |
| review* |
| “systematic review” |
| meta-analys?s |
| synthes?s |

**Table S2: Inclusion & exclusion criteria**

| **Inclusion criteria** | **Exclusion criteria** |
| --- | --- |
| - Systematic review with or without meta-analysis. - Report of the association or consequences of EDs or ED risk factors and mental disorders (e.g. depression, anxiety, substance use disorders). - Published in English language and peer-reviewed journal within the last 5 years (i.e Jan 2015). - Study conducted worldwide involving human participants such as children, adults were included. | - Conference abstracts narrative or literature reviews without quality appraisals of individual studies. - Literature review, scoping review, and narrative review was excluded. |

**Table S3: The Joanna Briggs Institute Critical Appraisal tools for use of JBI Systematic Reviews-Questionnaires**

|  |  |  |  |  |  |  |  |  |  |  |  |  |  |
| --- | --- | --- | --- | --- | --- | --- | --- | --- | --- | --- | --- | --- | --- |
|  | 1. Is the review question clearly and explicitly stated? | 2. Were the inclusion criteria appropriate for the review question? | 3. Was the search strategy appropriate? | 4. Were the sources and resources used to search for studies adequate? | 5. Were the criteria for appraising studies appropriate? | 6. Was critical appraisal conducted by two or more reviewers independently? | 7. Were there methods to minimize errors in data extraction? | 8. Were the methods used to combine studies appropriate? | 9. Was the likelihood of publication bias assessed? | 10. Were recommendations for policy and/or practice supported by the reported data? | 11. Were the specific directives for new research appropriate? | Score out of total of 11 | Percentage |
| ÁlvarezRuiz et al. 2015 | Yes | Yes | Unclear | No | Unclear | Unclear | Yes | Yes | Unclear | No | Yes | 5 | 45 |
| Baskin & Galligan 2019 | Yes | Yes | Yes | Yes | Yes | Yes | Unclear | Yes | Unclear | Yes | Yes | 9 | 81 |
| Conti et al. 2017 | Yes | Yes | Yes | Yes | No | Yes | Unclear | Yes | Unclear | Yes | Yes | 8 | 72 |
| Cucchi et al. 2016 | Yes | Yes | Yes | Yes | No | Yes | Yes | Yes | Unclear | No | Yes | 8 | 72 |
| Drakes et al. 2021 | Yes | Yes | Yes | Yes | Yes | Yes | No | Unclear | Unclear | Yes | Yes | 8 | 72 |
| Farstad et al. 2016 | Yes | Yes | Yes | Yes | Yes | Unclear | Unclear | Yes | Unclear | No | Yes | 7 | 63 |
| Fornaro et al., 2021 | Yes | Yes | Yes | Yes | Yes | Unclear | Yes | Unclear | No | Yes | Yes | 8 | 72 |
| Goldstein & Gvion, 2019 | Yes | Yes | Yes | Yes | No | No | Unclear | Yes | No | Yes | Yes | 7 | 63 |
| Kaisari et al. 2017 | Yes | Yes | Yes | Yes | Yes | Yes | Yes | Yes | No | Yes | Yes | 10 | 90 |
| Kerr-Gaffney et al. 2018 | Yes | Yes | Yes | Yes | Yes | Unclear | Yes | Yes | Yes | No | Yes | 9 | 81 |
| Levin & Rawana, 2016 | Yes | Yes | Yes | Yes | Yes | Unclear | Yes | N/A | No | Yes | Yes | 8 | 72 |
| Lloyd et al. 2019 | Yes | Yes | Yes | Yes | Yes | Yes | Yes | N/A | Yes | No | Yes | 9 | 81 |
| Mandelli et al., 2020 | Yes | Yes | Yes | Yes | Unclear | Yes | Yes | Unclear | Yes | Yes | Yes | 9 | 81 |
| Miller et al., 2022 | Yes | Yes | Yes | Yes | Yes | Unclear | Unclear | Unclear | No | Yes | Yes | 7 | 63 |
| Nazar et al. 2016 | Yes | Yes | Yes | Yes | Yes | Yes | Yes | Yes | Yes | Unclear | Yes | 10 | 90 |
| Nicholls et al. 2016 | Yes | Yes | Yes | Yes | Yes | Yes | Yes | N/A | Unclear | No | Yes | 8 | 72 |
| Puccio et al. 2015 | Yes | Yes | Yes | Yes | Yes | Unclear | Unclear | Yes | No | No | Yes | 7 | 63 |
| Silva et al. 2019 | Yes | Yes | Yes | Yes | Yes | Yes | Unclear | Unclear | Unclear | No | Yes | 7 | 63 |

**Average quality assessment score for all reviews: 72%**
